# Notes and News

**Published:** 1895-11-09

**Authors:** 


### NOTES AND NEWS.

(Collected and Communicated.)

HOSPITAL MEETINGS, &c.

St. George Hospital.—Quarterly General Court on Friday, November 8th, at 1 o'clock at the hospital.

Cholera is regarded with so much alarm in Europe that the appearance of a few isolated cases in any community creates little short of a panic. Yet in Burmah the average number of deaths from the disease during the last ten years has amounted to 5,000, and no special alarm is evinced by the fact.

The International Sleeping Car Company are now making their winter arrangements for the transit of invalids and others southwards. Four special services are arranged, which comprise the Calais-Mediterranean Express, the Mediterranean Express, the Nice Express, and the Rapide. The two last run from Paris.

The Birmingham Hospital Sunday Fund is now proceeding. The returns so far received show that this year's collection is smaller than that of last year. On this occasion the General Hospital will be benefited, for the Birmingham Fund arranges that the General Hospital, the Queen's Hospital, and the smaller charities of the city shall receive the collection alternately. The General Hospital will find contributions especially acceptable just now, as it is proceeding with its new buildings, and the work required at its hands has in no way diminished.

The action of the managers of the Bristol Children's Hospital in arranging a display at the Handicrafts Exhibition in Bristol has been very strongly condemned. Surgical appliances and bandaged dolls formed part of the exhibition, whilst nurses were in attendance primarily, though ultimately they were withdrawn. We have always warmly advocated the system of encouraging the public to see for themselves the work done at our hospitals, but the public exhibition of nurses and their appliances, even for the benefit of hospitals, is a thing greatly to be deprecated.

An American journal divides doctors into three classes—those who read, those who do not read, and those who pretend to read.

The Hartford Medical Society, United States, are now able to claim the legacy of 20,000 dollars left by Mrs. Mary Hunt, in memory of her husband, to erect a building for the use of the society. The condition attached, which was that a site free of incumbrance should be acquired, has been fulfilled.

The sixth Italian Congress of International Medicine closed on October 25th after having held the most successful meeting. Professor Baccelli read the opening address. Serum therapeutics were referred to in several connections, notably by Professor Maragliano in reference to his researches in tuberculosis.

The Poplar Hospital for Accidents will, we hope, receive a substantial addition to its funds through the opening of the Blackwall Tunnel on Saturday. This tunnel is the greatest river subway in the world. On Saturday the public were permitted to use it on payment of a small fee. Thousands of persons availed themselves of the opportunity of visiting so interesting an exhibition of engineering skill. Bands and processions assembled in honour of the occasion, and lent brightness and colour to the scene.

The Accident Insurance Company, St. Swithin's Lane, E.C., have taken a new departure by issuing a comprehensive policy providing for compensation in the event of accident or disease. Many who have not seen the wisdom of taking out policies covering accident alone may readily avail themselves of the increased advantages now offered, and so insure themselves against the risks to which all are liable. The company, it may be noted, continues the business founded in 1849. It is therefore the oldest of the Accident companies, and we have ascertained that the business of late has much improved.

The intention of erecting for the celebrated Danish Dr. Vilh. Meyer a monument in the city of Copenhagen, a movement which has been started outside Denmark, has called forth an unexpected squabble among some of the Copenhagen doctors. A score of well-known doctors and physicians, most of them men of undoubted standing, thought fit to publish a protest against a Meyer monument being erected in some public spot in Copenhagen. They pointed out the anti-Danish sentiments shown by Meyer during the first Prusso-Danish war (1848-50), and, secondly, mentioned that the significance of Meyer's scientific work and discoveries hardly justified such an exceptional honour. This protest has been almost universally condemned; it has called forth weighty counter protests from various sides, and a number of doctors and other distinguished citizens have hastened to swell the list of contributors to the Meyer monument fund. There can be no doubt that the twenty discontented gentlemen have attained exactly the opposite result of what they intended with their protest, which has only served to bring Dr. Vilh. Meyer's claims to the contemplated flattering tribute to his memory into bolder relief.

